# Bark beetle outbreak enhances biodiversity and foraging habitat of native bees in alpine landscapes of the southern Rocky Mountains

**DOI:** 10.1038/s41598-020-73273-z

**Published:** 2020-10-02

**Authors:** Thomas Seth Davis, Paul R. Rhoades, Andrew J. Mann, Terry Griswold

**Affiliations:** 1 Forest and Rangeland Stewardship, Colorado State University, Fort Collins, CO USA; 2 Idaho Department of Agriculture, Coeur d’Alene, ID USA; 3 Department of Plant Pathology, University of Minnesota, Saint Paul, MN USA; 4 USDA-ARS Pollinating Insects Research Unit, Utah State University, Logan, UT USA

**Keywords:** Forest ecology, Community ecology, Biodiversity

## Abstract

Landscape-scale bark beetle outbreaks alter forest structure with direct and indirect effects on plants and animals in forest ecosystems. Using alpine spruce forest and a native bee community as a study system, we tested how tree mortality from bark beetles impacts bee foraging habitats and populations. Bees were collected across the growing season (early-, middle-, and late-season) for two years using passive trapping methods, and collections were used to analyze patterns in species abundances and diversity. Three important findings emerged: (1) forest stands that were post-outbreak had 62% higher floral density and 68% more floral species during peak bloom, respectively, than non-affected stands; (2) bee captures were highest early-season (June) and were not strongly affected by bark beetle outbreak; however, mean number of bee species and Shannon–Weiner diversity were significantly higher in post-outbreak stands and this effect was pronounced early in the growing season. Corresponding analysis of β-diversity indicated higher accumulation of bee biodiversity in post-outbreak stands and a turnover in the ratio of *Bombus*: *Osmia*; (3) bee captures were linked to variation in foraging habitat, but number of bee species and diversity were more strongly predicted by forest structure. Our results provide evidence of increased alpine bee biodiversity in post-outbreak stands and increased availability of floral resources. We conclude that large-scale disturbance from bark beetle outbreaks may drive shifts in pollinator community composition through cascading effects on floral resources, mediated via mortality of overstory trees.

## Introduction

Bark beetle outbreaks are a central factor driving large-scale changes in structure, function, and composition of forest landscapes in western North America and in temperate conifer forests globally^1–3^. The view of the general public and many ecosystem management practitioners holds that bark beetle outbreaks are associated with negative impacts on forest ecosystems and that control efforts should be implemented to improve forest health^4,5^. However, recent work from European forest systems indicate that bark beetle outbreaks may enhance forest insect biodiversity with beneficial consequences for natural resource conservation^6^.

In the southern Rocky Mountain region extensive forest mortality has occurred during the past decade in high-elevation alpine Engelmann spruce (*Picea engelmannii* Parry ex Engelm.) stands due to a widespread outbreak of North American spruce beetle (*Dendroctonus rufipennis* Kirby), an aggressive phloem-feeding bark beetle species that colonizes live host trees and is a primary agent of tree death when population densities are high. Some reports estimate losses of ~ 20% forest cover in the state of Colorado alone due to tree mortality^7,8^, which could have considerable impacts on plants and animals that rely on alpine spruce forest for habitat. A recent regional study indicates that alpine spruce forest harbors a relatively unique community of native bees with many high-elevation specialists that can maintain viable populations even in extreme habitats^9^. Given recent concerns over loss of pollinator species due to land-use change and fragmentation^10^, it is vital to understand how very large disturbances such as spruce beetle outbreaks might impact pollinators and the resources they use.

Although the residual effects of spruce beetle-driven canopy mortality on forest bee and flowering plant communities are not particularly well understood, tree mortality could positively impact site occupancy by native species through a variety of mechanisms including indirect effects. For instance, reduced leaf area in post-disturbance stands can drive increased light penetration to the forest floor and subsequent recruitment of flowering plants^11,12^. Elucidating whether similar patterns occur following bark beetle outbreaks will be important for generating hypotheses about the impacts of outbreaks on ecosystem services and providing new tools to forest managers.

**Figure 1:**
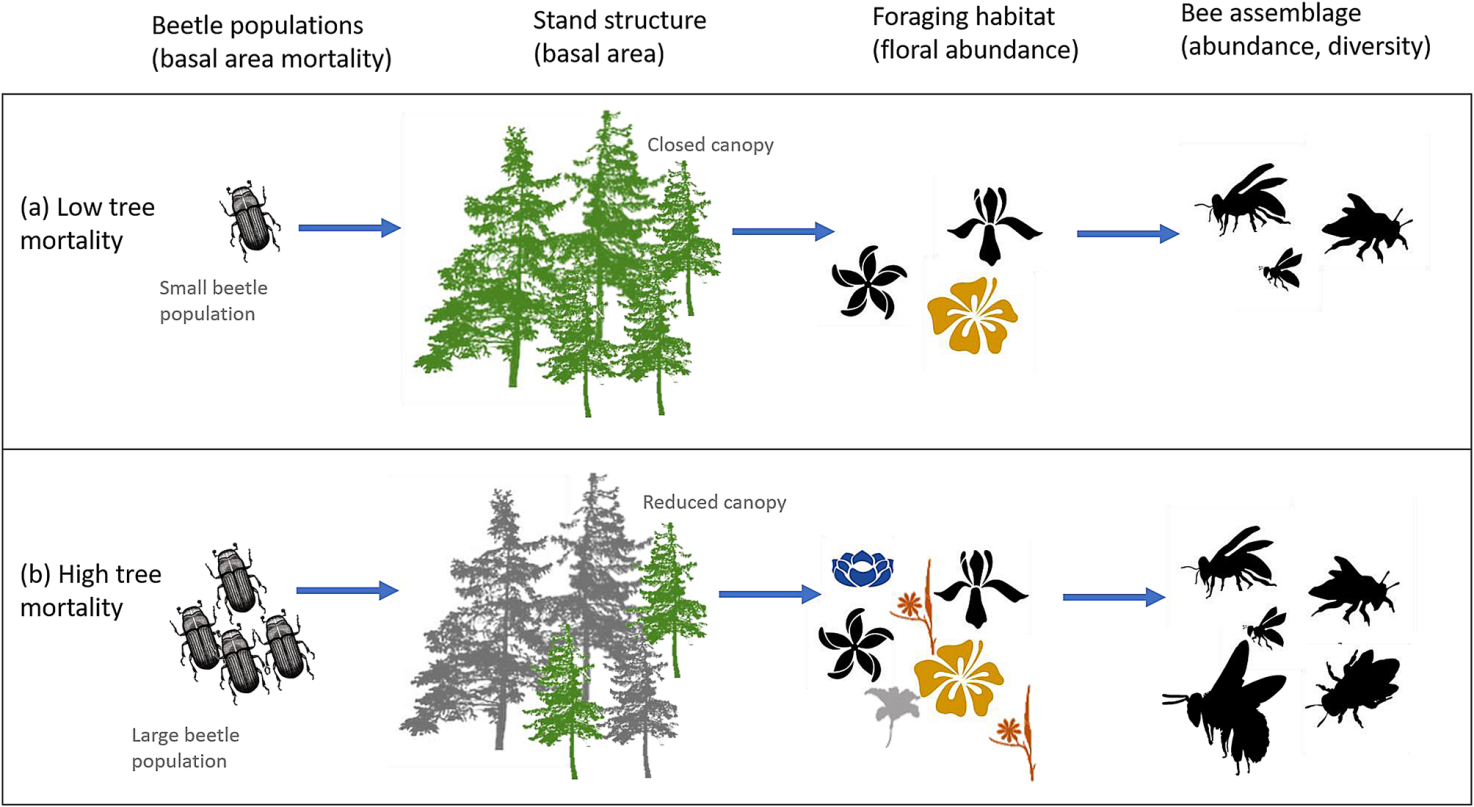
Conceptual representation of the hypothesized relationship between bark beetle outbreaks, forest structure, bee foraging habitat, and bee species assemblages. When forest mortality from spruce beetle is low (**a**), a closed canopy persists with relatively sparse floral resources and may be associated with a reduced bee species assemblage; but in post-outbreak stands that have experience considerable overstory mortality (**b**) there is an understory plant response that may be associated with improved foraging habitat and a larger and more abundant bee species assemblage. In the present study, elements of forest structure, foraging habitat, and bee communities were measured in non-affected and post-outbreak stands.

We test the hypothesis that spruce beetle outbreak-driven variation in forest structure affects floral resources and native bee assemblages in a southern Rocky Mountain landscape (Fig. 1). Utilizing a recent large regional outbreak as a natural experiment, our specific objectives were to (1) characterize the effects of spruce beetle outbreak on elements of forest structure and bee foraging habitat; (2) quantify how bee species abundance and diversity vary between stands affected by spruce beetle outbreak and non-affected stands; and (3) compare the relative effects of forest structure and foraging habitat on bee assemblages. Our studies link mortality of a dominant forest tree species from a widespread biotic agent of ecosystem disturbance to alpine bee biodiversity, with consequences for the interpretation of key forest disturbance processes and the conservation of ecosystem services.

## Methods and materials

**Figure 2:**
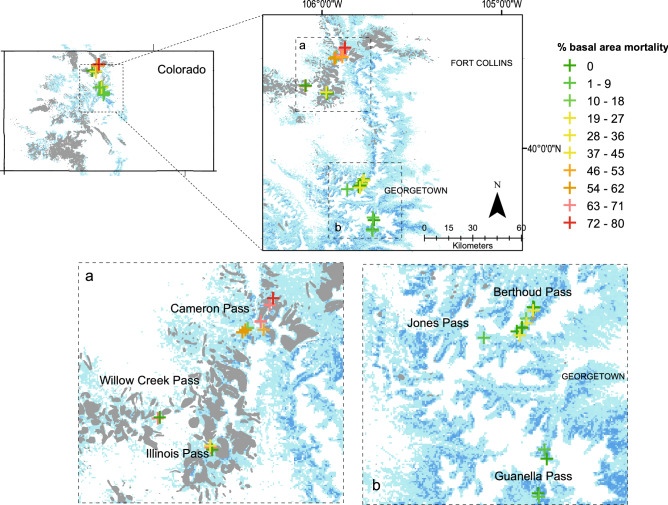
Map of the study landscape across six mountain pass regions within Colorado. Gray polygons denote aerially-mapped observations of spruce beetle mortality from 2010 to 2017, and crosshatches denote pollinator collection sites and plot locations. Crosshatches are color-coded to denote percent overstory basal area mortality. Blue shading shows the density of Engelmann spruce forest on the landscape. (https://www.esri.com/en-us/arcgis/about-arcgis/overview).

Adult bees were collected from a total of twenty-eight alpine sites (2800–3400 m elevation) in north-central Colorado during the growing seasons of 2018 and 2019 using passive trapping methods (Fig. 2) (ARC GIS, Esri, Inc., Redlands, CA, USA). Overstory vegetation in stands selected for study was predominately Engelmann spruce and subalpine fir (*Abies lasiocarpa*), but lodgepole pine (*Pinus contorta*) and quaking aspen (*Populus tremuloides*) were also occasionally present (46 and 7% of sites, respectively). Sites were chosen to represent either (1) forest stands that had experienced significant mortality of mature Engelmann spruce from spruce beetle (proportion of overstory basal area mortality ranged from 43 to 89%; n = 13) or (2) forest stands that had little or no evidence of contemporary overstory tree mortality (proportion of overstory basal area mortality ranged from 0 to 28%; n = 15). Initial site selection of spruce beetle-affected locations was achieved using data from federal aerial detection surveys^13^ and was standardized to reflect spruce stands with mapped spruce beetle-driven tree mortality 5–10 years prior to the time of the study (i.e., overstory mortality was recorded by surveyors between 2009 and 2013).

In each selected stand, site-level information on forest structure was collected on 0.01 ha fixed-area plots (10 m^2^). Bee passive traps (described below) were subsequently placed in the center of fixed area plots at each collection period to associate vegetation measurements with bee assemblages. On each plot, trees were censused and their diameter at breast height (dbh) recorded; species was also recorded for each tree as well as mortality status (alive or dead). From these data the proportion of dead overstory basal area (%) was computed and treated as a variable for analysis, as well as other basic forestry measurements including forest basal area (m^2^/ha^−1^), stand quadratic mean diameter (cm), and tree density (trees/ha^−1^).

In addition to measuring elements of forest structure, elements of bee foraging habitat, including mean floral resource abundance and number of species, were also determined for each site in July, the period when most forb species at alpine spruce forest sites are actively blooming in northern Colorado^14^. Floral data were collected in each study year using five replicate 1 m^2^ quadrats. One quadrat was placed at the center of each site directly under the trapping location, and remaining quadrats were placed at a distance of 2 m in each cardinal direction. Within each quadrat each individually distinguishable inflorescence in flower at the time of survey was considered as a single tally, and plant genus or species was recorded. Inflorescence abundance and tallied floral species values were treated as a subsample and averaged together from the five quadrats at each site to produce an estimate of mean floral abundance and mean number of species at peak bloom for all trapping sites in each collection year.

Previous studies indicate that bee communities in alpine spruce forest are predominantly *Bombus* spp. but also vary during the course of the growing season^9^; accordingly, each site was sampled three times in each year of the study to reflect major periods of bee activity during the growing season, including (1) June 15–20, ‘early-season’; (2) July 15–20, ‘mid-season’; and (3) August 18–22, ‘late-season’ (28 sites × 3 collection periods × 2 years = 168 collections total). At each collection period, a blue vane trap (Springstar, Inc., Woodinville, WA, USA) was hung from existing vegetation at a height of ~ 1 m for 72 h, and traps were fitted with a wire mesh insert to reduce the probability of inundation with water^9^. Upon collection trap contents were euthanized by placing on dry ice and returned to the lab where all bees were pinned and identified to the lowest taxonomic level possible. In most cases this was to genus and species, but some specimens could only be sorted to morphospecies (denoted ‘sp 1’, ‘sp 2′, etc.). Voucher specimens are curated at the C.P Gillette Museum of Arthropod Diversity at Colorado State University.

### Data analyses

All statistical analyses were implemented using the R programming language (V.3.5.1, “Feather Spray”^15^), and unless otherwise stated incorporate a Type I error rate of α = 0.05 for assigning statistical significance to modeled effects. However, effects that were significant at the α = 0.10 were interpreted as ‘marginally significant’ in order to account for factors that may be ecologically important but were not classically ‘significant’ due to the high variability often present in ecological studies. For parametric tests, assumptions of homoscedasticity were verified prior to analysis using Levene’s test^16^.

### Characterize the effects of spruce beetle outbreak on elements of forest structure and bee foraging habitat

To compare forest overstory vegetation structure between spruce stands affected by spruce beetle outbreak and non-affected stands, a two-sample Student’s t-test was used to compare mean basal area, average quadratic mean diameter, mean tree density, and the mean proportion of basal area mortality between ‘spruce beetle-affected’ (n = 13) and ‘non-affected’ stands (n = 15). Mean floral abundance and number of species were also compared between spruce beetle-affected and non-affected stands using an ANOVA model that treated year as a random effect. For both tests, normality of test distributions was verified by visual inspection of histograms.

### Quantify bee species abundance and diversity in post-outbreak and non-affected stands

Since bee communities tend to vary across the growing season, a two-factor ANOVA was used to analyze the fixed effects of seasonality (Jun, Jul, Aug), outbreak status (spruce beetle-affected and non-affected stands), and the seasonality × outbreak status interaction on bee captures, number of bee species, and the Shannon-Weiner diversity index (H′ statistic); sample year was incorporated as a random effect. This analysis used site × month × year observations as the unit of replication (n = 168). Bee capture data were log-transformed to conform to assumptions of normality prior to analysis. Since Shannon’s H′ cannot be computed when no species are present (H′ = 0 when only a single species is present), collections that resulted in no bee specimens (~ 30% of collections resulted in no captures) were necessarily omitted from consideration when analyzing model effects on Shannon’s H′. However, 0 values were incorporated into analyses on bee captures and number of species.

Bee species β-diversity across spruce beetle-affected and non-affected stands was analyzed by generating sampling curves^17^ using the ‘iNEXT’ package^18,19^. Estimates were interpolated from sample-based abundances to account for different numbers of bee captures and extrapolated to 2 × the size of the smallest sample^20^ (q = 0). Community composition was also compared between spruce beetle-affected and non-affected stands using a distance-based framework. A species-abundance matrix of bee captures from all sites (rows = sites, columns = bee species counts) was transformed into a matrix of Bray–Curtis dissimilarities and effects of site status (i.e., spruce beetle-affected or non-affected) on community composition were analyzed using the ‘adonis2’ function (permutational multivariate analysis of variance^21^, n permutations = 9999) in the R add-on package ‘vegan’ V2.5-5^22^. Results were visualized using non-metric multidimensional scaling (NMDS), produced with the ‘metaMDS’ function in package ‘vegan’.

### Compare relative effects of forest structure and foraging habitat on bee assemblages

A generalized linear model framework (family: gaussian, link function: identity) was used to determine and compare the relative effect sizes of forest structure variables and foraging habitat variables on bee assemblages, treating individual sites (n = 28) as the unit of analysis. Since forest structural measurements (tree density, basal area, basal area mortality, and quadratic mean diameter) did not differ between years and therefore only a single observation was available for each site, multi-year observations including floral surveys and bee specimen collections were averaged across years to produce site-level mean values. Prior to interpreting a regression model, correlation analysis was performed on independent variables and highly correlated variables (≥ 0.60 correlation coefficient) were omitted from analysis (Supplementary Fig. S1). Accordingly, independent variables used in the model were tree density, basal area, basal area mortality, and mean number of floral species. Dependent variables included mean bee captures, mean number of species, and mean Shannon–Weiner diversity; both independent and dependent variables were normalized to (µ = 0, σ = 1) prior to analysis.

## Results

### Characterize the effects of spruce beetle outbreak on forest structure and bee foraging habitat

**Figure 3:**
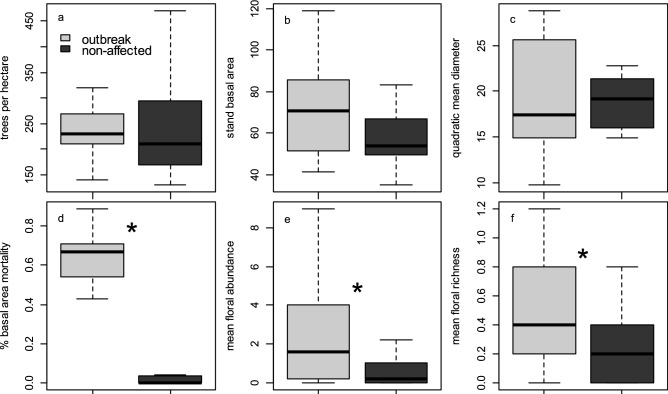
Distribution of stand structural attributes including (**a**) tree density, (**b**) forest basal area (m^2^ ha^−1^), (**c**) quadratic mean diameter (cm), and (**d**) proportion of basal area mortality in spruce stands that experienced spruce beetle outbreak and non-affected stands; distributions shown in (**a**)–(**d**) include both living trees and dead trees. Elements of foraging habitat including (**e**) floral abundances and (**f**) number of floral species (per m^2^) were also quantified, and asterisks denote statistically significant differences (*P* < 0.05) between means.

Characteristics of overstory vegetation in spruce beetle-affected and non-affected stands were similar and verified that site selection reflected stands which were structurally similar with the exception of the proportion of basal area killed by spruce beetle 5–10 years prior. Tree density did not differ significantly between affected and non-affected stands (affected stands = 235.3 ± 24.5 trees per ha, non-affected stands = 252.6 ± 22.8 trees per ha; *t*_26_ = 0.515, *P* = 0.610, Fig. 3a); neither did stand basal area (affected stands = 76.4 ± 8.2 m^2^/ha, non-affected stands = 64.21 ± 7.6 m^2^/ha; *t*_26_ = 1.085, *P* = 0.287, Fig. 3b) nor quadratic mean diameter (affected stands = 20.4 ± 1.4 cm, non-affected stands = 18.6 ± 1.3 cm; *t*_26_ = 0.882, *P* = 0.385, Fig. 3c). However, the mean proportion of basal area mortality was 62.3 ± 4.0% in spruce beetle affected stands and 6.6 ± 3.8% in non-affected stands, and this difference was statistically significant (*t*_26_ = 9.988, *P* < 0.001, Fig. 3d).

**Figure 4:**
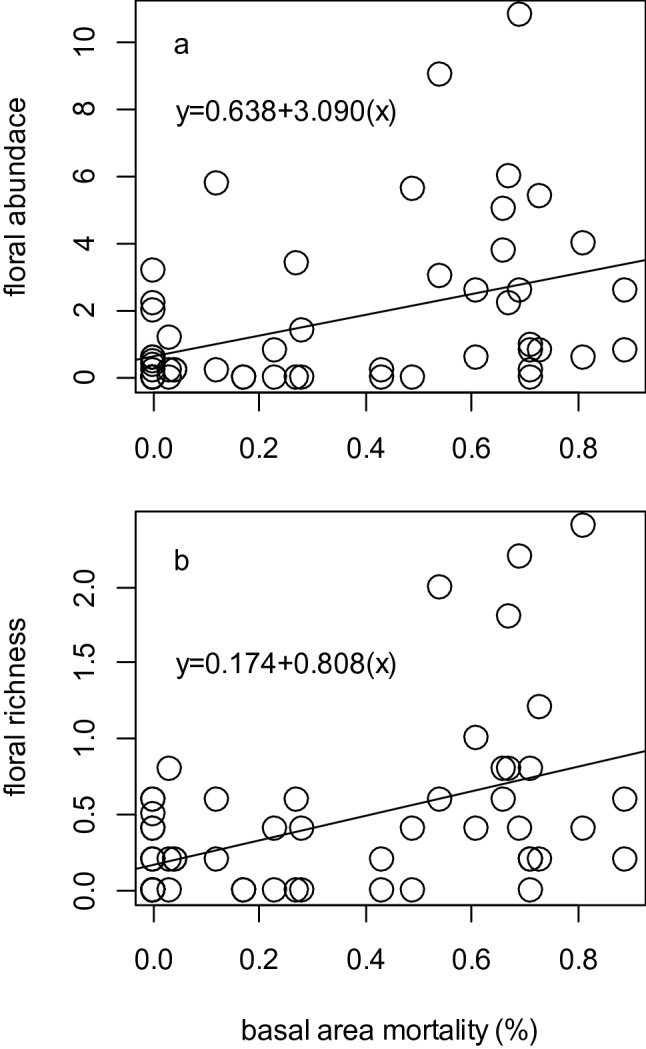
Relationship between proportion of basal area mortality and (**a**) mean floral abundance and (**b**) number of species per m^2^ in alpine Engelmann spruce forest stands. Both regression models are significant at a Type I error rate of α = 0.05.

In addition to stand structural attributes, elements of bee foraging habitat quantified during the peak bloom period varied between stands affected by spruce beetle outbreak and non-affected stands. Floral taxa typical of surveyed stands included *Aquilegia coerulea*, *Trollius laxus*, *Erythronium grandiflorum*, *Calpyso bulbosa*, *Noccaea fendleri*, *Swertia radiata*, *Mertensia* spp., *Lupinus* spp., and especially *Arnica* spp. and *Vaccinium* spp. Mean floral abundance was 67% higher in spruce beetle-affected stands than in non-affected stands (whole model: *F*_2,51_ = 4.819, *P* = 0.012, Fig. 3e); year-to-year variation had little effect on floral abundances and only accounted for only ~ 3% of modeled variance (Table 1; computed as SS_year_/SS_model_). Similarly, mean number of floral species was 66% higher in spruce beetle-affected stands than in non-affected stands (*F*_2,51_ = 7.680, *P* = 0.001); however, there was a small effect of year-to-year variation on number of floral species which accounted for ~ 8% of modeled variance (Fig. 3f; Table 1). Subsequent least-squares linear regression analysis revealed a significant positive association between the proportion of basal area mortality in spruce stands and floral abundance (*F*_1,52_ = 10.243, *P* = 0.002, Fig. 4a) and number of floral species (F_1,52_ = 13.528, *P* < 0.001, Fig. 4b), with basal area mortality explaining a moderate proportion (16–20%) of variance in floral resources.

**Table 1:** ANOVA table summarizing differences in mean floral abundances and floral species richness between spruce beetle-outbreak and non-affected stands (‘site status’), in the context of year-to-year variation.

Variable	Source	SS	df	F	*P*
Mean floral abundance/m^2^	Site status (outbreak vs. non-affected)	38.337	1	7.628	0.008
	Year (random)	10.102	1	2.010	0.162
	Error	256.307	51	–	–
Mean number of floral species/m^2^	Site status (outbreak vs. non-affected)	2.535	1	10.125	0.002
	Year (random)	1.317	1	5.235	0.026
	Error	12.768	51	–

### Quantify bee species abundance and diversity in post-outbreak and non-affected stands

Overall γ-diversity and captures of alpine bees during the study period was represented by 789 specimens (Table 2) that comprised 13 genera and 45 species. Two genera, *Bombus* (Apidae) and *Osmia* (Megachilidae), comprised a majority of captures and accounted for 71 and 23% of the total sample, respectively. Three species were dominant in the sample: *Bombus mixtus* Cresson (26% of total sample), *B. flavifrons* Cresson (20% of total sample), and *Osmia bucephala* Cresson (10% of total sample).

**Table 2:** Summary of alpine bee γ-diversity from twenty-eight sample sites distributed across six mountain pass regions in northern Colorado during the study period (2018 and 2019 growing seasons).

Family	Genus	Species	Captures
Andrenidae	*Andrena*	*padoucorum*	1
		sp 1	1
Apidae	*Anthophora*	*bomboides*	4
		*montana*	3
		sp 1	1
		*terminalis*	1
		*urbana*	1
	*Bombus*	*appositus*	7
		*balteatus*	34
		*bifarius*	40
		*centralis*	3
		*fernaldae*	6
		*flavifrons*	162
		*frigidus*	1
		*huntii*	20
		*insularis*	1
		*melanopygus*	81
		*mixtus*	207
		*occidentalis*	4
		*sylvicola*	1
	*Diadasia*	sp 1	1
	*Melissodes*	sp 1	3
Colletidae	*Hylaeus*	sp 1	1
Halictidae	*Dufourea*	sp 1	1
	*Lasioglossum*	*sisymbrii*	1
		sp 1	7
Megachilidae	*Coelioxys*	sp 1	1
	*Hoplitis*	sp 1	3
	*Megachile*	*melanophaea*	1
		sp 1	8
		*townsendiana*	1
	*Osmia*	*aff. pusilla*	13
		*albolateralis*	13
		*brevis*	4
		*bucephala*	79
		*iridis*	11
		*paradisica*	20
		*pentstemonis*	22
		*simillima*	5
		*sladeni*	1
		sp 1	9
		sp 2	1
		sp 3	2
		*tanneri*	2
		SUM	789

Specimens grouped to distinct morphospecies but that were not identifiable to species level are identified as ‘sp’.

**Figure 5:**
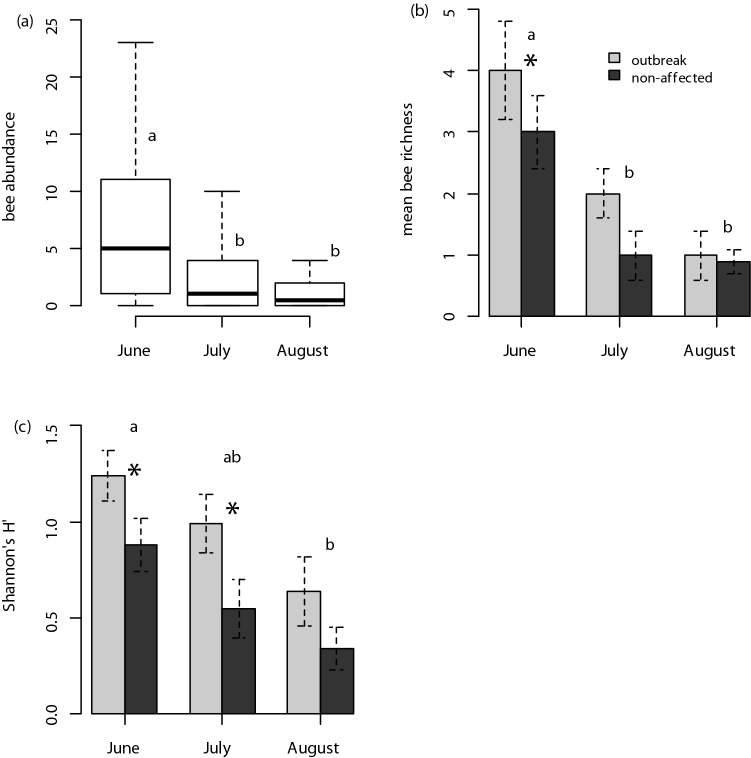
(**a**) The distribution of bee captures in vane traps relative to collection month (seasonality). Mean (**b**) number of bee species and (**c**) and Shannon–Weiner diversity viewed across both seasonality and stand status (outbreak vs. non-affected stands). In (**b**) and (**c**) error bars are ± 1 SE and asterisks (*) denote the main effect of outbreak status; lettering denotes the main effect of seasonality in all three figures.

Although mean bee captures were 22% higher in stands affected by spruce beetle outbreak than in non-affected stands, variation in bee captures was high and mean captures did not vary due to an effect of spruce beetle outbreak (*F*_1,153_ = 2.531, *P* = 0.105). In contrast, there was significant variation in mean bee captures due to an effect of seasonality (*F*_2,153_ = 16.967, *P* < 0.001), and mean bee captures were 55 and 65% higher in June than in July and August, respectively (Fig. 5a). However, there was no evidence of variation in mean bee captures due to an outbreak status × seasonality interaction (*F*_2,153_ = 0.296, *P* = 0.742). Year effects were minimal and differences between sample years only accounted for a small fraction (0.1%) of modeled variance in captures.

On average, number of bee species was 37% higher in spruce beetle-affected stands than in non-affected stands, and this effect was significant (*F*_1,153_ = 6.299, *P* = 0.013). There was also a significant effect of seasonality on the number of bee species (*F*_2,153_ = 15.402, *P* < 0.001) and mean number of bee species was 53 and 71% higher in June than in July and August, respectively (Fig. 5b). However, there was no effect of an outbreak status × seasonality interaction on mean number of bee species (*F*_2,153_ = 0.752, *P* = 0.473). Again, year effects accounted for only a small fraction (~ 0.01%) of the modeled variance in number of bee species.

Spruce beetle outbreak also impacted bee diversity; mean Shannon-Weiner diversity (as measured by Shannon’s H′ statistic) was 42% higher in stands affected by spruce beetle outbreak than in non-affected stands, and this difference was statistically significant (*F*_1,91_ = 12.442, *P* < 0.001). Mean Shannon–Weiner diversity also varied significantly due to a seasonal effect and was 25 and 52% higher in June than in July and August, respectively (*F*_2,91_ = 6.216, *P* = 0.004; Fig. 5c). In contrast, there was no evidence that an outbreak status × seasonality interaction affected bee diversity (*F*_2,91_ = 0.060, *P* = 0.941). As with captures and number of species, the effects of year-to-year variation on diversity were minimal and accounted for only 1% of the modeled variance.

**Figure 6:**
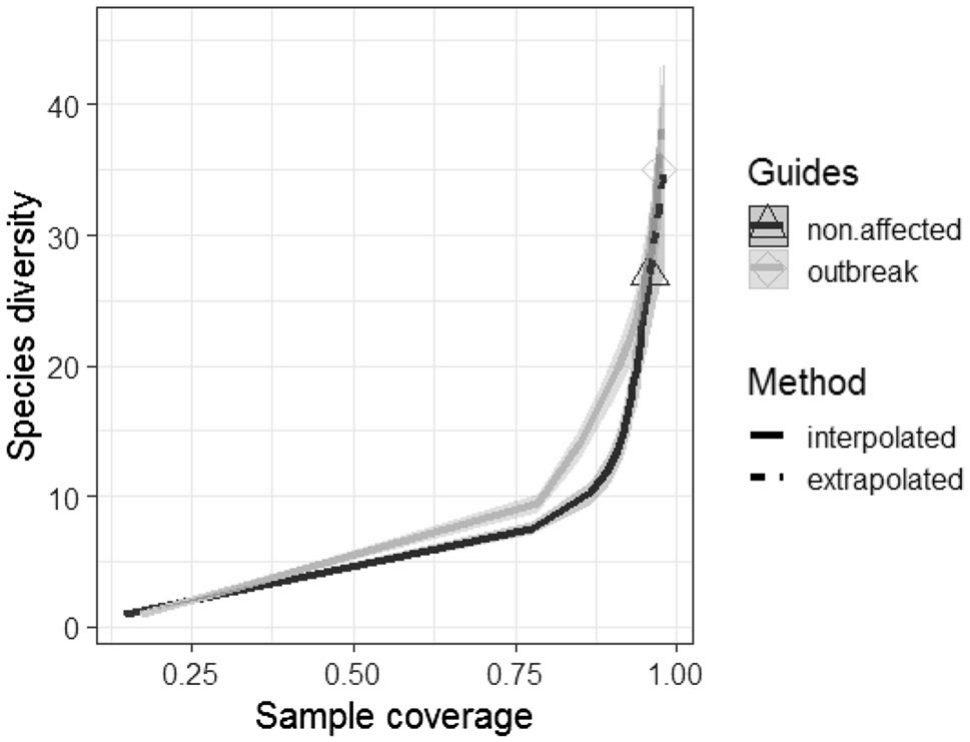
Sample-based accumulation of bee species diversity in alpine forest stands with significant overstory mortality from spruce beetle (‘outbreak’, light gray) and stands that were non-affected by spruce beetle (‘non-affected’, dark gray line). Shading shows the bootstrap-estimated 95% confidence interval of each sampling curve.

**Figure 7:**
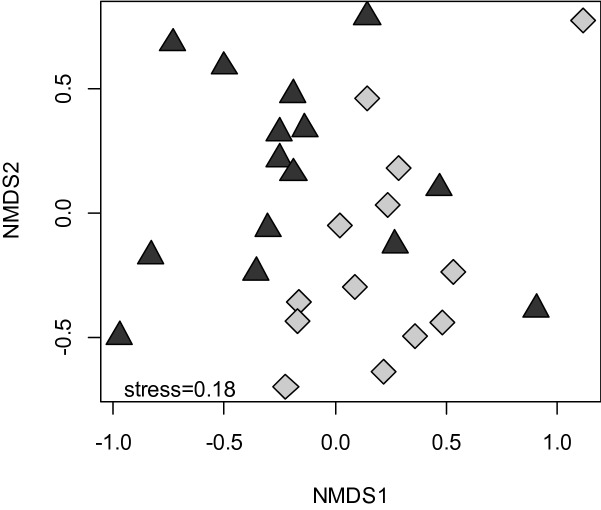
Ordination of bee community assemblages (NMDS), compared between post-outbreak (light gray diamonds) and non-affected stands (dark gray triangles).

**Figure 8:**
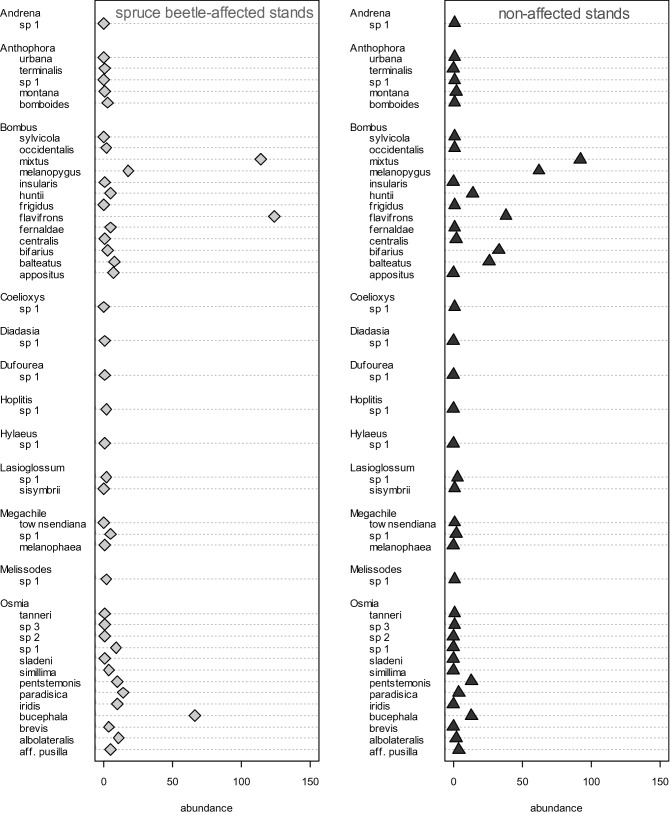
Comparison of turnover in bee species abundances (grouped by genus) between post-outbreak and non-affected stands.

Analysis of β-diversity using rarefaction curves coupled with bootstrapped confidence intervals indicated that accumulation of bee biodiversity was generally higher in stands affected by spruce beetle outbreak than in non-affected stands (Fig. 6). In addition, the species composition of bee community assemblages differed significantly between post-outbreak stands and non-affected stands (*F*_1,26_ = 3.478, *P* < 0.001, Fig. 7). This difference was primarily driven by the ratio of *Bombus* and *Osmia* in collections, with the frequency of *Osmia* spp. increasing by ~ 20% on average in spruce-beetle affected stands (χ^2^ = 47.664; df = 11, *P* < 0.001; Fig. 8).

### Compare relative effects of forest structure and foraging habitat on bee assemblages

Bee captures were positively associated with increasing number of floral species (β = 0.488, *P* = 0.040) but not with any modeled forest structure variables. In contrast, mean number of bee species was marginally negatively associated with increasing tree density (β =  − 0.339, *P* = 0.083). Similarly, Shannon-Weiner diversity was marginally negatively associated with increasing forest basal area (β =  − 0.353, *P* = 0.085) and negatively associated with increasing tree density (β =  − 0.420, *P* = 0.030). Neither mean number of bee species nor diversity were significantly associated with modeled elements of foraging habitat (number of floral species, Table 3).

**Table 3:** Summary of a generalized linear model analysis to describe variation in bee assemblages due to effects of forest structure and foraging habitat.

Response variable	Parameter	Estimate (β)	SE	SS	df	*t*-score	*P*
Bee captures	Intercept	0.000	0.180	0.000	1	0.000	1.000
	Tree density	− 0.174	0.185	0.710	1	− 0.939	0.357
	Basal area	− 0.204	0.201	0.602	1	− 1.020	0.319
	% Basal area mortality	− 0.121	0.239	0.616	1	− 0.504	0.619
	**# Floral species**	**0.488**	**0.224**	**4.289**	**1**	**2.179**	**0.040**
	Residual variance	**–**	**–**	20.784	23	**–**	**–**
Number of bee species	Intercept	0.000	0.181	0.000	1	0.000	1.000
	**Tree density**	− **0.339**	**0.187**	**3.243**	**1**	− **1.815**	**0.083**
	Basal area	− 0.187	0.202	0.181	1	− 0.926	0.364
	% Basal area mortality	0.169	0.241	1.772	1	0.700	0.491
	# Floral species	0.190	0.226	0.650	1	0.841	0.409
	Residual variance	–	–	21.151	23	–	–
Shannon–Weiner diversity	Intercept	0.000	0.176	0.000	1	0.000	1.000
	**Tree density**	− **0.420**	**0.181**	**4.154**	**1**	− **2.315**	**0.030**
	**Basal area**	− **0.353**	**0.196**	**2.127**	**1**	− **1.803**	**0.085**
	% Basal area mortality	0.145	0.234	0.763	1	0.618	0.542
	# Floral species	0.067	0.219	0.080	1	0.305	0.763
	Residual variance	–	–	19.874	23	–	–

Significant (*P* < 0.05) and marginally significant (*P* < 0.10) effects are highlighted in bold text.

## Discussion

Our results suggest that shifts in forest structural characteristics (tree mortality) from bark beetle outbreaks in high-elevation spruce-fir forests have impacts on both bee foraging habitats (Fig. 4) and bee species assemblages, and we report evidence that significant overstory tree mortality is likely to result in an overall increase in bee captures and changes in both Shannon-Weiner diversity and β-diversity in post-outbreak stands (Figs. 5, 6, 7, 8). Our study focused on characterizing bee assemblages in stands that were between 5 and 10 years post-outbreak and stands that have not been recently affected by bark beetles, so the duration of these effects in post-outbreak stands remains unknown and merits additional investigation. Mortality of forest basal area from spruce beetle was positively correlated with floral abundance and number of floral species, and understory forb responses to canopy gaps created by overstory tree mortality in post-outbreak stands is likely a mechanism by which bee communities respond to bark beetle outbreaks (Figs. 1, 3, 4). Although enhanced floral species richness in post-outbreak stands was associated with an increase in bee captures (Table 3), variation in site-level forest structural characteristics were more strongly related to mean number of bee species and diversity, indicating the possibility of differential controls over bee abundance and diversity. In addition, effects of bark beetle outbreaks on bee assemblages were strongly seasonal with differences in number of bee species and diversity observed primarily in early-season collections—but by late in the growing season bee diversity metrics were comparable between post-outbreak and non-affected stands (Fig. 5). This effect is due in part to increased site occupancy by *Osmia* spp. (a genus that is typically active early in the growing season) in post-outbreak stands. These collective findings have implications for interpreting the ecological effects of landscape-scale forest disturbances, and indicate that bark beetle outbreaks can contribute to variation in bee assemblages across alpine landscapes^4^.

Our collection details the bee fauna present across alpine tree line sites in Colorado, for which little empirical data are available. Despite two years of trapping effort overall bee captures and γ-diversity in high-elevation alpine spruce-fir forest were relatively low in comparison with other recent studies of forest bees in similar systems. For example, Rhoades et al.^9^ collected approximately 16% more specimens using similar methods in a mixed conifer forest in one trapping season, though the present study reports a higher overall number of species. Here, most sample locations were near alpine tree line and mean site elevation was 3200 m (~ 10,550 ft); in contrast, bee assemblages in Rhoades et al.^9^ were sampled at an elevation of 2800 m (~ 9,240 ft). This comparison suggests that in montane landscapes moderate increases in elevation (400 m) are likely to be associated with considerable reductions in bee abundances^23^ and very high-elevation sites present challenging conditions that may be beyond the thermal tolerances of most species^24,25^.

Nonetheless, there was evident turnover in bee species between post-outbreak and non-affected stands: in post-outbreak stands *Osmia* spp. (mason bees, Megachilidae) represented ~ 31% of bee captures but comprised only ~ 11% of captures in non-affected stands (Fig. 8). This difference is consistent with the life history of *Osmia*—many species rely on cavities in wood or woody debris for nesting habitat^26^—and overstory tree mortality from bark beetles likely results in a considerable increase in available woody material for use as nesting resources. For instance, Klutsch et al.^27^ projected that regional mountain pine beetle (*D. ponderosae*) outbreaks are likely to result in increases of downed woody debris by up to 80% in post-outbreak stands. In the present study, most trees killed by spruce beetle persisted as standing snags, which contain numerous bark crevices, branch and wood splits, and wood borer or woodpecker holes^28^ that could be exploited by cavity-nester^29^. Moreover, standing dead trees often decay at a slower rate than trees which have fallen and spruce wood is particularly resistant to mineralization following tree death^30^, potentially indicating that standing dead Engelmann spruce trees provide long-term increases in available nesting spaces for mason bees. In addition to *Osmia* spp., bumblebees (*Bombus* spp*.*) were abundant in our study and are often among the most common taxa found at high-elevation sites^31,32^, due in part to their relatively high thermal mass in comparison to other bees^24,25,33^. Our collections provide evidence of shifts in *Bombus* assemblages following bark beetle outbreak: *B. flavifrons* was substantially more abundant in post-outbreak stands, whereas several high-elevation specialists were more frequent in non-affected stands including *B. balteatus* and *B. melanopygus*. This turnover in species composition could suggest that certain bee species are well-adapted to exploit the conditions created by bark beetle outbreaks. Potential mechanisms underlying this variation are unexplored but could relate to shifts in nesting habitats (e.g., coarse woody debris) in addition to changes in foraging habitats or preferences.

A comparison of several recent studies and the current report reveals a growing body of evidence that both natural and managed ecosystem disturbances are important for maintaining biodiversity of native bee populations across coniferous forest landscapes. For example, complementary findings from Burkle et al.^32^ and Galbraith et al.^20^ indicate that wildfire disturbances are associated with increased diversity of multiple bee communities and across multiple conifer forest cover types. In addition to effects on taxonomic diversity, bee functional trait variation both within and between species are also responsive to these disturbances^32^. Similarly, studies from boreal forests in Europe report that prescribed fires in Scots pine (*Pinus sylvestris* L.) forests result in distinct bee assemblages with improved pollination services^34^, and most solitary bees are not predicted to be directly negatively affected (e.g., by mortality from soil heating) during fire disturbances^35^. In the present study, sampling efforts were focused primarily on high-elevation, non-managed forest systems where the structural effects of bark beetle outbreaks may persist for long periods. However, forest management efforts that change elements of forest structure, particularly overstory cover, also impact bee communities. For instance, recent work from southeastern U.S. forests found that restoration-based canopy thinning resulted in higher abundance and richness of native bees^36^, and in boreal forests where clearcutting practices are common removal of the overstory can positively impact forb recruitment and site occupancy by native bee assemblages^37^.

We report a similar pattern of increased forb and bee abundance and diversity following an extensive overstory tree mortality event driven by tree-killing bark beetles (*Dendroctonus rufipennis*). As in Rhoades et al.^9^, we found that the number of bee species and bee species diversity generally declined as forest basal area and tree density increased. These elements of forest structure (basal area and tree density) were similar between Engelmann spruce-subalpine fir stands that were 5–10 years post beetle outbreak and non-affected stands, however, both basal area and tree density could decline by ~ 65% on average once standing snags fall (Fig. 3)—leading to additional light availability at the forest floor with potential consequences for forb recruitment. Although fall rates of beetle-killed Engelmann spruce have not been definitively determined, earlier studies report that over 80% of trees remain standing up to 25 years post-outbreak^38^. Standing snags in our study had experienced complete needle-fall and most have lost fine or tertiary branches (similar post-outbreak conditions reported by Raphael and Morrison^39^), indicating that photosynthetic conditions in the understory likely differs considerably between the post-outbreak and non-affected stands despite similar basal area. This is consistent with the finding that mean inflorescence densities were higher in post-outbreak stands, where approximately 28% more bees were collected.

However, several limitations of the present study should be considered when interpreting our results. First, we only consider a single window of ‘time since outbreak’, grouping sites which were between 5- and 10-years post-mortality. Second, our sampling of floral resources was on a relatively small scale (site-level, with a total of 5 m^2^ sampled for inflorescences at each site × year) in comparison to the effect of the disturbance we were interested in (bark beetle outbreak, which occurs across large landscapes). Accordingly, future work in this system could improve upon our findings by considering a wider post-disturbance time series and better scaling the sampling of floral resources to the disturbance event, which would help to determine longevity of the effects that we report here and could further refine our understanding of cascading effects of bark beetle outbreaks on organisms important for forest ecosystem function. Third, we employ a single method for capturing bees, which can impact the overall assemblage that is sampled. For example, Rhoades et al.^40^ examined variation in bee captures between blue vane traps (used here), colored pan traps, and aerial netting and discovered that although there was considerable overlap in terms of community assemblage sampled among the three methods, there were also some bee species that tended to be idiosyncratic to specific collection methods. However, that study also revealed some sampling advantages to blue vane traps; namely, blue vane traps captured the greatest bee abundance and species richness of all the methods. Other authors have suggested that passive trapping methods are preferable when sampling large landscapes, as traps are not susceptible to individual observer skill or experience as is the case with manual netting^41^.

Across many ecosystems, bark beetle outbreaks are drivers of landscape-scale spatial heterogeneity across large areas and contribute to the structural complexity of forest systems^42,43^. Structural heterogeneity and complexity are strong predictors of gene flow and biodiversity in bee communities^43,44^ and various authors have concluded that a landscape mosaic of disturbances (and varying disturbance severity) that results in a range of structural classes enhances collective pollinator richness, ecosystem function, and ecosystem services^32,45–48^. Among ecosystem managers, as well as the general public, bark beetle outbreaks are typically considered as undesirable disturbances for which prevention strategies should be sought^5^, but our study suggests that outbreaks may help to create habitat for pollinator communities in wilderness settings. Alpine bees may be especially vulnerable to shifts towards homogenous ecosystem structure due to their relatively less abundant and diverse assemblages in comparison with lower-elevation conifer forest types. Accordingly, we conclude that bark beetle outbreaks in high-elevation forests should be considered as important for promoting forb recruitment and site occupancy by native bee populations. Follow-up work to experimentally link outbreak-driven structural heterogeneity across forest landscapes with site-level pollination services would be especially informative for understanding relationships between bark beetle outbreaks and ecosystem function.

## Supplementary information

Supplementary Information.
