# Supplementary material for: Bark beetle outbreak enhances biodiversity and foraging habitat of native bees in alpine landscapes of the southern Rocky Mountains
Source: Sci Rep. 2020 Oct 2;10:16400. doi: 10.1038/s41598-020-73273-z (PMC7532438; doi:10.1038/s41598-020-73273-z)
Supplement: Supplementary file 1 — Supplementary Information. [file 41598_2020_73273_MOESM1_ESM.pdf]

Supplementary material for *Scientific Reports*

Bark beetle outbreak enhances biodiversity and foraging habitat of native bees in an alpine landscape

Thomas Seth Davis[1]*, Paul R Rhoades[2], Andrew J Mann[3], Terry Griswold[4]

[1]Forest and Rangeland Stewardship, Colorado State University, Fort Collins, CO, USA

[2]Idaho Department of Agriculture, Coeur d'Alene, ID, USA

[3]Department of Plant Pathology, University of Minnesota, Saint Paul, MN, USA

[4]USDA-ARS Pollinating Insects Research Unit, Utah State University, Logan, UT, USA

*Correspondence: email: seth.davis@colostate.edu

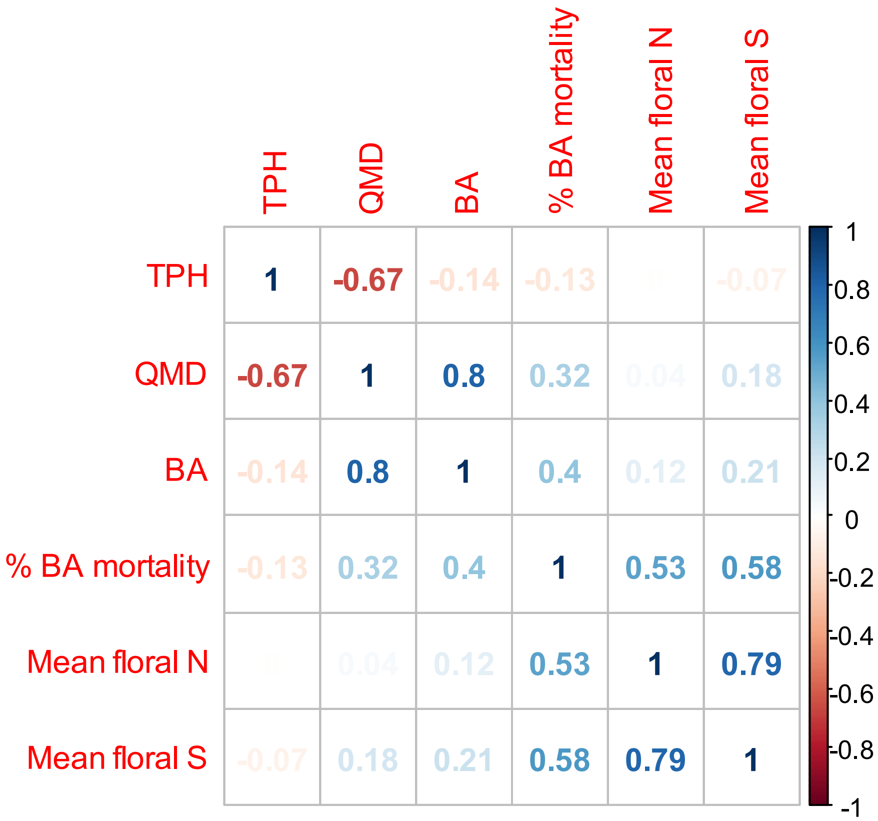

Figure S1. Correlation analysis of the relationship between candidate independent variables related to forest structure and foraging habitat in alpine Engelmann spruce stands; values shown in the matrix are Pearson correlation coefficients. Variables with correlations ≥0.60 were omitted from multiple regression analysis; accordingly, both QMD and mean floral abundance were selected for omission to reduce correlation structure in the model. Abbreviations are as follows: TPH, tree per hectare; QMD, quadratic mean diameter; BA, basal area; N, abundance; S, species richness.
